# Printed, flexible, compact UHF-RFID sensor tags enabled by hybrid electronics

**DOI:** 10.1038/s41598-020-73471-9

**Published:** 2020-10-06

**Authors:** Carol L. Baumbauer, Matthew G. Anderson, Jonathan Ting, Akshay Sreekumar, Jan M. Rabaey, Ana C. Arias, Arno Thielens

**Affiliations:** 1 Berkeley Wireless Research Center, EECS Department, UC Berkeley, Berkeley, 94704 CA USA; 2 Present Address: Waves Research Group, Ghent University/imec, Ghent, 9052 Belgium

**Keywords:** Electrical and electronic engineering, Mechanical engineering

## Abstract

Sensor data can be wirelessly transmitted from simple, battery-less tags using Radio Frequency Identification (RFID). RFID sensor tags consist of an antenna, a radio frequency integrated circuit chip (RFIC), and at least one sensor. An ideal tag can communicate over a long distance and be seamlessly integrated onto everyday objects. However, miniaturized antenna designs often have lower performance. Here we demonstrate compact, flexible sensor tags with read range comparable to that of conventional rigid tags. We compare fabrication techniques for flexible antennas and demonstrate that screen and stencil printing are both suitable for fabricating antennas; these different techniques are most useful at different points in the design cycle. We characterize two versions of flexible, screen printed folded dipoles and a meandered monopole operating in the 915 MHz band. Finally, we use these antennas to create passive sensor tags and demonstrate over the air communication of sensor data. These tags could be used to form a network of printed, flexible, passive, interactive sensor tags.

## Introduction

Radio Frequency Identification (RFID) is a technology that enables wireless short- and medium- range tracking and identification of objects. Wireless communication takes place between a central transceiver, or reader, and a simple transponder, or tag, which is attached to an object of interest. RFID tags can be active, with an on-board power supply such as a battery; or passive, harvesting their energy from the RF signal sent by the reader. A tag capable of sensing some characteristic of its environment and relaying that sensor data in addition to its identification information is known as a sensor tag. RFID systems can be designed to operate in a variety of frequency bands, depending on the transmission distance and data rate required for the application. Ultra-high frequency RFID (UHF-RFID), defined by the EPC Gen2 standard, uses the unlicensed band in the 915 MHz range in the United States. RFID sensor tags consist of an antenna, a radio frequency integrated circuit (RFIC)^1^, and at least one sensor.

Historically, most of the UHF-RFID tags were only used for identification^2^. The first efforts to develop UHF-RFID tags with sensor abilities used (de-)tuning of the antenna as a method for sensing^3–5^, changing the electrical properties of the tag^6,7^, or circuits external to the RFIC with or without an external micro-controller mounted on flexible substrates or PCBs^8–11^. A next generation of RFID sensing tags used single-chip configurations without external micro-controllers^12–17^. The main advantages of these configurations is that they can be powered wirelessly via the RFID technology, the sensor data can be transmitted digitally over the air, they require fewer components, and can be more compact than configurations with external circuits.

The ability to wirelessly communicate with sensor tags can be used for wearable health monitoring devices^18–20^, medical applications^21^, environmental monitoring^22^, smart packaging^23^, and vehicular technology^24^. RFID tags are expected to have major impact in the transport of perishable goods^25^, supply-chain management^26^, and warehouse management^27^, especially for high-value products. Dynamic inventory tracking and multi-user quality control could benefit from a network of nodes which only requires the central reader to have an energy source. Passive tags, with a variety of sensors, can allow for distributed, mobile, and interactive sensing.

**Figure 1:**
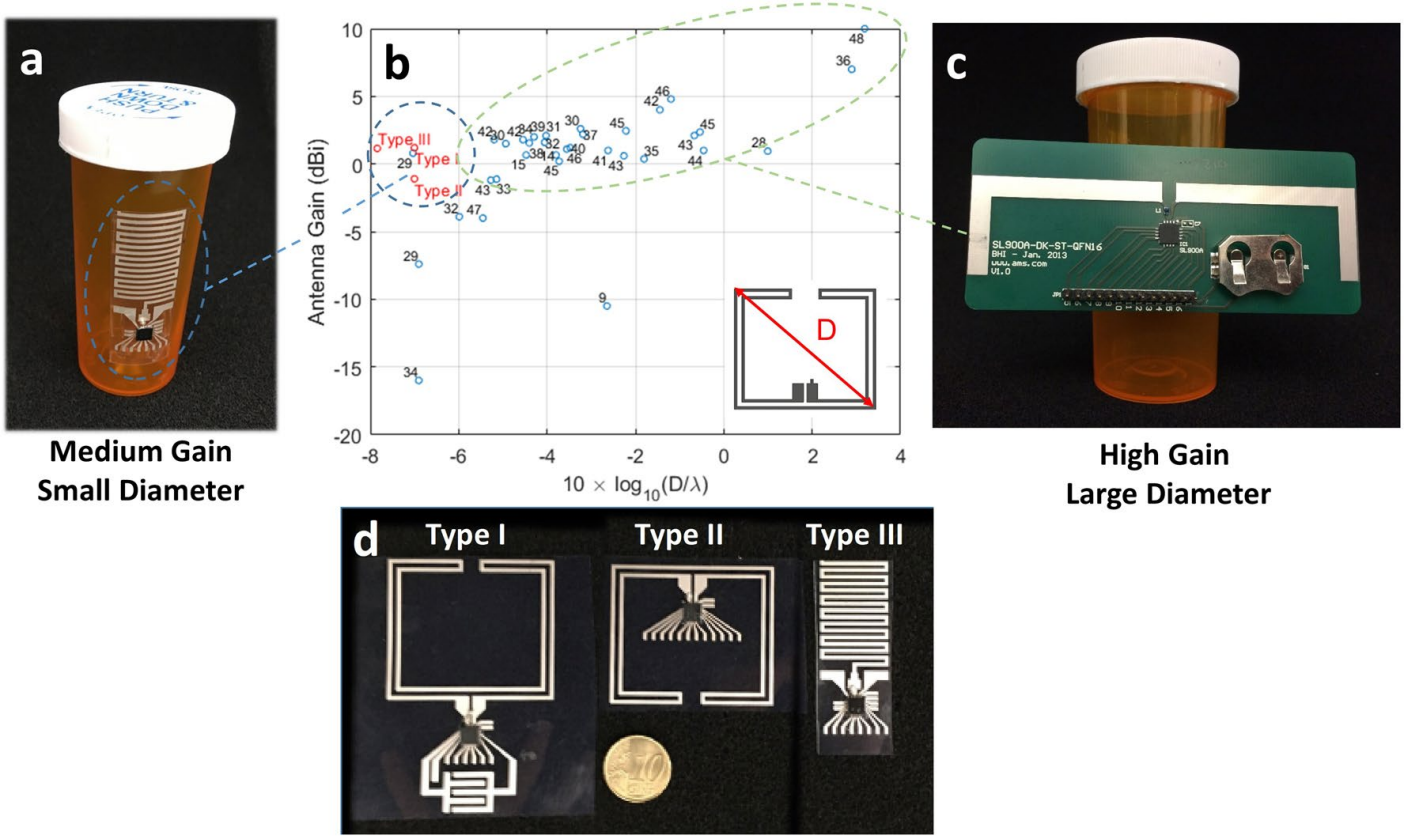
An overview of the printed tags presented in this manuscript in comparison to the state of the art. (**a**) A printed, flexible, meander monopole antenna can be used in a passive UHF RFID tag that is easily integrated onto a small, non-conformal package like a pill bottle. (**b**) Printed antennas on a flexible substrate reported in literature (blue circles) can have high to moderate antenna gain. However, such antennas also have large diameter relative to their operating wavelength (D/$\lambda$), where “diameter” refers to the longest distance between two points on the antenna, as illustrated in the inset. This makes them less suitable for applications where small form-factors are desirable. Other examples in literature that pursued a smaller D/$\lambda$ resulted in antennas with low gains (bottom of the figure). Notably, the antennas described in this work (red circles) have small D/$\lambda$ and moderate gain. (**c**) A typical dipole antenna with large D is not suited for integration on small packages. (**d**) This work presents three types of printed, passive tags on flexible substrate for operation in the UHF RFID band (902–928 MHz). The antennas used in Type I are externally-fed folded dipoles, Type II are internally-fed folded dipoles, and Type III are on meander monopoles with ground pads on the front side of the substrate. All tags operate using an RFIC mounted on printed traces.

Figure 1a illustrates how a compact, flexible RFID tag can be integrated onto the packaging of sensitive, high-value goods, for example in the pharmaceutical industry. Because the RFIC is small, the antenna becomes the limiting factor in the size and flexibility of the tag. However, small antennas typically have much poorer performance than their larger counterparts. Figure 1b shows that in literature, antennas with small longest linear dimension typically also have reduced maximum antenna gain^9,14,15,28–48^. Antenna miniaturization is an area of antenna design which aims to reduce the size of an antenna while maintaining acceptable performance^49^. This reduction in size can be of the longest linear dimension, *D*, the area, or the volume of the antenna. For RFID sensor tags that will be integrated with everyday objects, the longest linear dimension is an important parameter. The meandered monopole antenna shown in Fig. 1a, was miniaturized by folding a long wire into a serpentine pattern. Figure 1c shows that a conventional dipole with long linear dimension cannot be easily attached to a small object like a pill bottle. The antennas designed and studied here have small linear dimensions while preserving reasonable gain, as shown in Fig. 1b.

A second important part of RFID tags is the RFIC. An RFIC is a silicon chip with radio transmitter circuitry, some read/write memory, and ports for external sensors. A passive, backscatter RFIC harvests its energy from the RF field of the reader and communicates by changing its impedance to modulate the amount of power it reflects back to the reader^1^. An RFIC can be paired with a sensor that can monitor a parameter of interest. Sensors can be on board the RFIC itself or fabricated separately and connected to the chip.

Each component of the sensor tag has unique requirements which can be addressed by a variety of fabrication techniques. In particular, the antenna will be relatively large (on the order of mm or cm), should be inexpensive to mass produce, and flexible so that it can be integrated onto a variety of common objects. Additive manufacturing techniques such as printing are well suited to meet these demands.

The RFIC should be small, and needs to have read/write memory and RF circuitry, both of which are best made with conventional silicon micro-fabrication techniques. Depending on the application space and sensor type, it might be advantageous to have either printed flexible sensors or on-chip sensors. The concept of flexible hybrid electronics (FHEs)^21,50^ merges these different fabrication techniques: each component is fabricated using the method that best meets its needs, and a functional system is created by integrating the components, some of which are flexible, and some of which are rigid. The RFID sensor tags presented here have printed, flexible antennas and touch sensors, which are combined with a rigid silicon RFIC and its temperature sensor.

In this work, we optimized the design of compact, printed, flexible antennas, and integrated these printed antennas with rigid RFICs to form tags.We have developed three types of tags to demonstrate that our design flow and fabrication procedure can be used for multiple tag designs. In addition, we demonstrated streaming temperature and interactive touch-sensor data from a network of passive nodes.

The novelties of this study are the following: first, we present printed antennas on a flexible substrate that provide a higher gain per maximal dimension D than what has been previously demonstrated in literature, see Fig. 1b. Second, one of these antennas is fabricated using multiple printing techniques, which allows for direct comparison of the printing techniques. Third, we fabricate three different antenna topologies using one printing technique, which demonstrates its usability. Fourth, we demonstrate that our tag can perform temperature measurements that are not influenced by humidity. This is a known disadvantage of flexible, printed temperature sensors. Finally, we demonstrate a wireless, flexible, printed capactive touch sensor that is interrogated using passive backscattering.

## Results

A fundamental component of the RFID tag is the antenna. Here, we describe antenna design, fabrication with several different printing techniques, and characterization. From the analysis of different printing techniques, a preferred prototyping technique was chosen, which was also used to prototype other geometries. Finally, screen-printed antennas of three designs were characterized.

Antenna design began with numerical simulations. An antenna type and dimensions were chosen based on the envisioned application, specifications on antenna parameters, and boundary conditions of the fabrication. Initial designs were proposed and simulated in an electromagnetic solver, which returns antenna parameters. The dimensions and feeding strategy of an antenna were adapted digitally in order to optimize antenna parameters and ensure compliance with the predetermined specifications. However, simulation results typically differ from reality because of assumptions and idealizations made in the calculations, so physical antennas were fabricated and characterized as part of the antenna design. With this information numerical models were validated.

The performance of a physical antenna depends on its fabrication method. Many methods of printing have been used to make RF systems and antennas^2,51^, including screen-printing^32,52^, gravure-printing^53^, spray-coating^29^ and inkjet-printing^3,8,54^. Using our first antenna design, Type I (see Supplementary Figure A.1a), we characterized the impact of printing technique on antenna performance by printing the same antenna with four different techniques: inkjet printing, stencil printing, spray coating, and screen printing. The printing technique impacted trace thickness, overall profile shape, edge and surface roughness, and sample-to-sample variability. These physical parameters in turn impacted the RF performance of the antenna.

**Figure 2:**
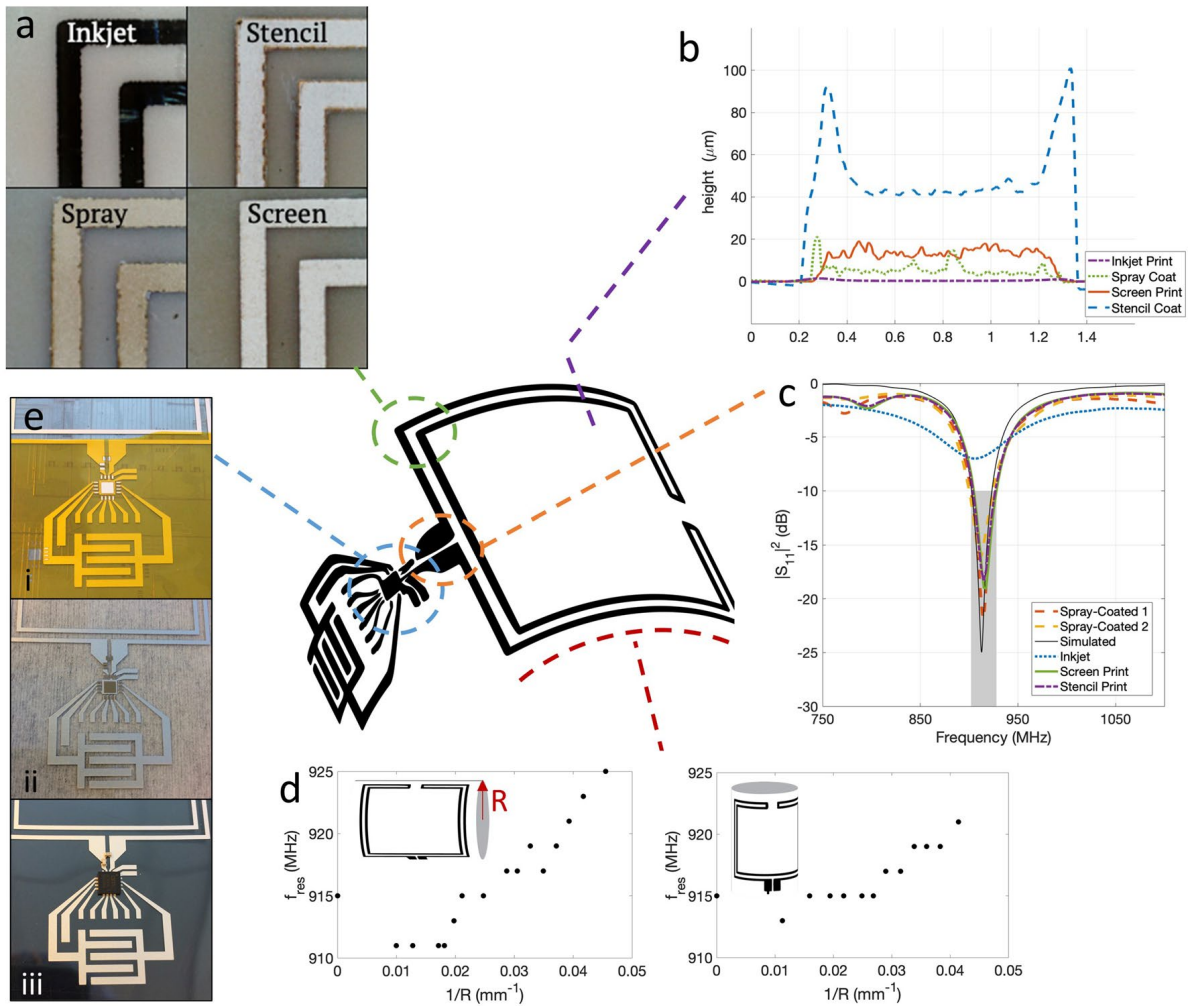
Physical and electrical characteristics of type I antennas and tags fabricated with different printing techniques. (**a**) Microscope images of the top-left corner of the antenna show surface and edge roughness of patterns made with inkjet, stencil, screen, and spray printing (in a clockwise direction). (**b**) Profiles of traces printed with the same four techniques. The vertical axis shows the trace height, while the horizontal axis shows the traces’ transverse direction. The colors indicate different printing techniques. (**c**) Power reflection coefficient (|S11|^2^) parameters show that spray coated, stencil printed, and screen printed samples achieve good resonance (|S11|^2^ $< -10$ dB) in the targeted 902-928 MHz band, while inkjet printed samples have a much smaller resonance peak. (**d**) Bending the antenna around a smaller radius shifts the resonance peak (frequency where S11 is the lowest) to higher frequencies. Up to radii of 20 mm, the peak stays within the 902–928 MHz band. (**e**) Rigid components are attached to to a screen-printed tag in three steps: (i) a laser cut stencil is used to define areas where (ii) conductive ink is applied with a razor blade. (iii) The components are placed with a pick-and-place tool.

Figure 2a shows the edge roughness of one corner of Type I antennas made with the different printing techniques. Inkjet printed traces had a smooth surface and periodic extensions along the edge. The regularly spaced bulges resulted from the spreading of regularly spaced ink droplets. The stencil and spray coated traces were defined by laser-cut adhesive stencils as described in the Methods section. The laser cutting of the stencils made rough edges which can be seen in both these sets of traces. The screen printed traces had comparatively straight edges.

The thickness profiles of traces made with these four techniques are shown in Fig. 2b. The inkjet printed traces were by far the thinnest, at about 200 nm thick. This is typical for inkjet-printed traces, which are made with relatively small volumes of low viscosity ink. The stencil printed samples had the thickest traces of 43 μm, while the spray-coated traces were about 3–8 μm thick, and the screen printed samples were about 20 μm thick. The large ridges on the edges of many stencil printed samples and some spray coated samples formed when ink dried on the edge of the tape stencil and remained after the stencil was removed. The surface roughness, quantified here as the root mean square error of the central portion of each trace, also depended on fabrication method. The surface roughness was 14 nm, 2.3 μm, 1.6 μm, and 2.0 μm for inkjet, spray, stencil, and screen printed traces respectively. Surface roughness has some impact on the impedance of printed RF components with lower roughness leading to lower Ohmic resistance^55^.

The thickness (*t*) of the traces significantly impacts their resistance and RF efficiency. Thin conductors are characterised by their sheet resistance ($R_s$), with $R_s=\rho / t$ [in $\Omega / \square ]$. The resistivity $\rho$ [in $\Omega m$] is a material property; in inks it is determined by the type of conductor (silver, for all of our inks), as well as the type and concentration of additives and binders in the ink, and the annealing conditions. The ink used for inkjet printing had the lowest $\rho =1.15\times 10^{-7}\Omega m$, while the resistivity of the ink used for screen and stencil printing was $\rho =2\times 10^{-7}\Omega m$ and that of the spray coating ink was $\rho =8.5\times 10^{-7}\Omega m$. However, because the inkjet-printed traces were much thinner than those obtained using any other techniques, the sheet resistance was highest for inkjet printing with $R_s=0.5 \Omega / \square$ in comparison to $R_s=1.8\times 10^{-2}\Omega / \square$ and $R_s=2.9\times 10^{-2}\Omega / \square$ for stencil and screen-printing, respectively.

For AC applications, comparing the skin depth ($\delta _s$) to trace thickness provides insights about the current distribution and losses in a thin trace. AC current travels along the edge of a conductor, and the skin depth gives an indication of the thickness of a conductive material that carries current. Skin depth is given by:

1 $$\begin{aligned} \delta _s=\frac{1}{\sqrt{(\pi f \mu \sigma )}} \end{aligned}$$

where $\sigma =\frac{1}{\rho }$ is the conductivity, $f=915 MHz$ is the frequency of interests, and $\mu =\mu _0=4\pi \times 10^{-7} [H/m]$ is the magnetic permeability^56^. A conductor whose thickness is less than $\delta _s$ will have significant losses compared to a thick conductor or an infinitely thin perfect conductor. At 915 MHz, using the inks’ resistivities, we found $\delta _s = 6~\mu m$ for inkjet-printed traces, $\delta _s = 15 ~\mu m$ for spray coated traces, and $\delta _s = 7~\mu m$ for stencil and screen printed traces, which were made with the same ink. The screen-printed and stencil printed traces had a thickness that exceeded their $\delta _s$, while the spray coated traces were somewhat thinner than their $\delta _s$, and the inkjet-printed traces were more than an order of magnitude thinner than $\delta _s$ in that material. This means that the inkjet printed trace suffered significant losses compared to the other printing techniques.

The physical properties of the printed samples influenced the RF performance of the printed antennas. We quantified the RF performance using three parameters: antenna power reflection coefficient ($|S_{11}|^2$), antenna impedance, and antenna directvitity/gain. The $|S_{11}|^2$ of the Type I printed antenna fabricated using different printing techniques were measured to characterize the RF performance of the antennas. $|S_{11}|^2$ is a standard performance metric for antennas^1^. At the resonant frequency small $|S_{11}|^2$ (large negative values in dB) indicate that most of the power provided to the antenna is not reflected but is transmitted. Our design targeted 90% accepted power in the UHF RFID frequency band, illustrated by a grey rectangle in Fig. 2c. We defined the bandwidth of an antenna as the range of frequencies over which the antenna complies with this design goal, i.e. it has an $|S_{11}|^2 \le -10~dB$. The resonant frequency of the antenna is that frequency where |S11|^2^ is minimal. Simulations of the Type I antenna had a resonance peak at 914 MHz and a bandwidth of 22 MHz, as shown in Fig 2c. The inkjet-printed samples did not meet the target of having a $|S_{11}|^2 \le -10~dB$ in the UHF-RFID band, due to their limited trace thickness. The stencil-printed samples had a resonance peak at $915\pm 3.5$ MHz and bandwidth of $23\pm 1$ MHz, while spray coated antennas had a peak at $913\pm 1$ MHz and 24 MHz bandwidth. Screen printed samples’ resonance was at $917\pm 2$ MHz with a bandwidth of $24 \pm 1$ MHz.

The effect of bending the flexible antennas was studied by bending the antenna in two directions, see Fig. 2d. We have verified that when the antenna was bent, its resonance frequencies increased, which is illustrated in Fig. 2d for bending around two orthogonal axes. Bending the antenna reduced its size, which increased the resonant frequency. Bending radii down to 24 mm for both axes did not shift the antenna out of the desired frequency band. Bending the antenna down to a radius of 24 mm shifted the resonance frequency by − 1% and 0.7%, respectively. Therefore, this antenna design is functional when the tag is flexed. The minimum bending radius was limited by the rigid SMA connector.

The reflected power measured by $S_{11}$ comes from impedance mismatch between the generator, cables, and the antenna. RF equipment is designed to have standard impedance of $50+0j\Omega$. A load perfectly matched to its feed line has impedance equal to the complex conjugate of the line, which is also $50+0j\Omega$. The reflection coefficient $S_{11}$ is a complex number which is related to the complex impedance of the antenna ($Z_A$) by:

2 $$\begin{aligned} S_{11}=\frac{Z_A-Z_L}{Z_A+Z_L} \end{aligned}$$

where $Z_L$ is the impedance of the feed line, 50 $\Omega$. From this, the impedance of the antennas was calculated^57^. Screen-printed samples ($N =8$) had a real impedance of $48\pm 3~\Omega$ at 915 MHz, while stencil-printed samples ($N=7$) had a real impedance of $51\pm 6.9~\Omega$ at the same frequency. These were also in good agreement with the simulated value of 50 $\Omega$. The inkjet-printed antennas had relatively large imaginary impedance ($Im(Z)=-50\pm 1.8~\Omega$, $N=4$), caused by the limited thickness of the traces.

Directivity describes how an antenna directs the power it radiates; it is defined as “the ratio of radiation intensity in a given direction to the radiation intensity averaged over all directions”^49^, which is commonly plotted on a polar plot or three dimensional rendering. If given as a single number, directivity refers to the maximum value. It is usually more practical to measure gain (G), which is the directivity times the radiation efficiency. Radiation efficiency is the ratio of power radiated by the antenna to power accepted by the antenna and accounts for losses in the antenna. Gain is given in units of dBi, where the “i” indicates the measurement is with respect to isotropic. An antenna with a gain of 0 dBi would radiate the same power in a given direction as an isotropically radiating source. A linear, half wavelength dipole orientated along the z-axis has maximum gain of 1.64 (2.15 dBi) in the x–y plain, and nulls along the z-axis^57^.

**Figure 3:**
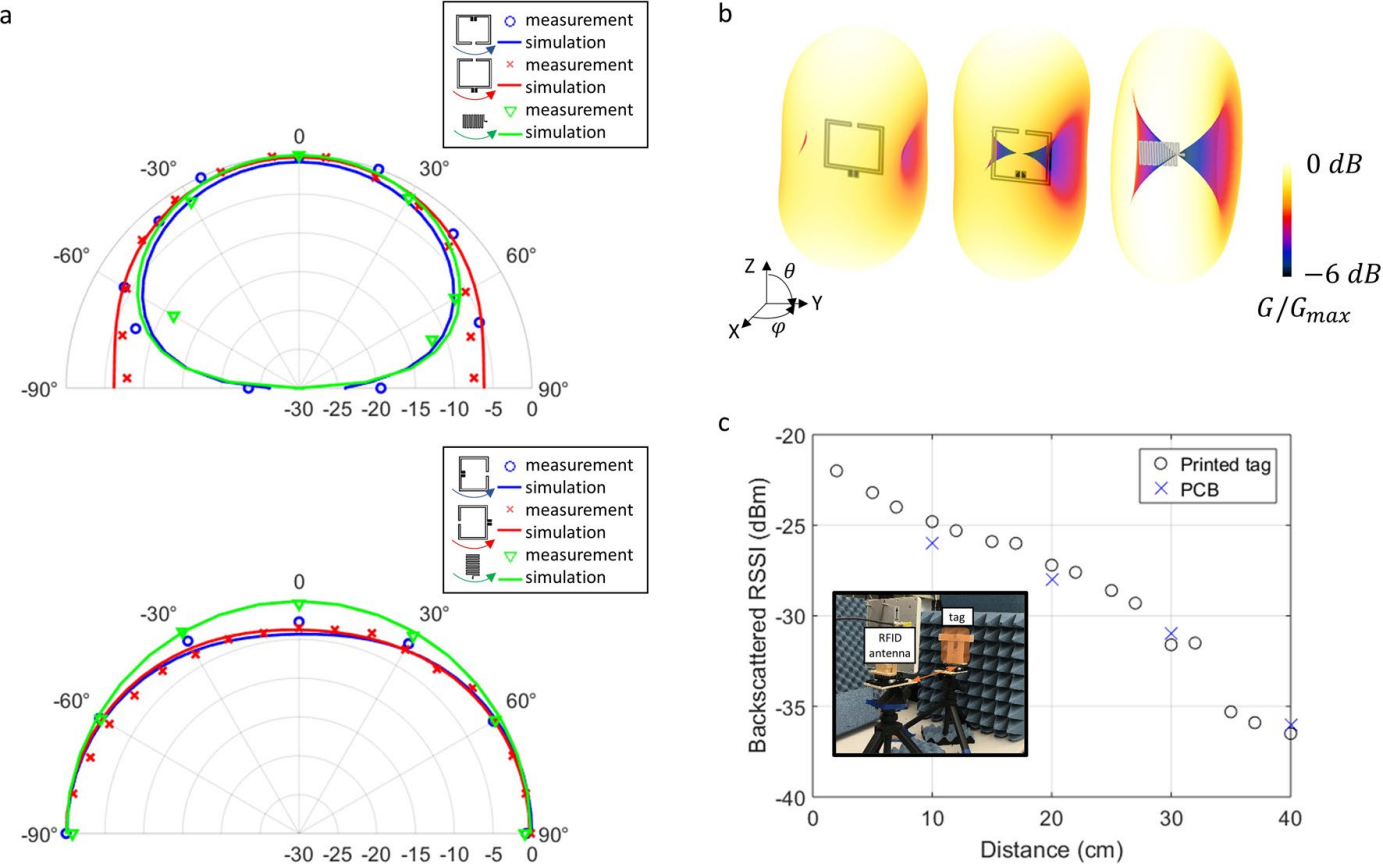
Wireless performance of the studied printed antennas and tags. (**a**) Simulated (solid line) and measured (discrete points) radiation patterns (in terms of antenna gain normalized to the maximum antenna gain) for each of the three types of antennas. (**b**) 3D representations of the simulated radiation patterns shown in (**a**). The colors indicate relative strength of the antenna gain. All antennas show desirable, dipole-like radiation patterns with uniform gain at $\phi = 0^{\circ }$ and varying elevation angle, as well as two nulls at $\theta = 90^{\circ }$ and varying azimuth (**c**) Back scattered Received Signal Strength Indicator (RSSI) as a function of distance for a printed type I tag and the conventional rigid PCB tag shown in Fig. 1c. The printed tag and rigid PCB have similar RSSI for read distances up to 40 cm, demonstrating the wireless functionality of the printed tags with significantly lower D/$\lambda$ (see Fig. 1b). The inset shows the measurement setup with the circularly polarized RFID reader antenna on the left and the tag under test on the right (indicated in orange). The separation distance (horizontal axis) is measured along the orange arrow.

We designed and characterized three types of antennas. Type I was a folded dipole which is fed from the outside of the antenna. Type II was a very similar folded dipole fed from inside the square. Type III was a meandered monopole, with small ground pads on each side of the monopole. We measured the gain patterns of each of the three antenna types by rotating one antenna along the azimuth and zenith angles and measuring power transmission from one antenna to the other, as described in the Methods section. Figure 3a shows the measurements of the antenna gain as a function of the azimuth angle (top) and zenith angle (bottom). The simulated and measured radiation patterns were very similar to those that are theoretically expected for a dipole antenna, although the folding slightly increased the gain in the plane of the print. The measured gain patterns showed relatively good correspondence with the simulated values, indicated by the solid lines. Figure 3b provides three dimensional renderings of the normalized gain of each antenna type. Again, these demonstrate the dipole-shaped radiation pattern of the antenna.

For each antenna type, we measured absolute antenna gain in the direction of maximum gain where possible, using screen printed samples. Type I had a simulated gain of 1.3 dBi, and we measured 1.2 ± 0.2 dBi. Type II antennas had a simulated maximum gain of 1.6 dBi, but we were unable to measure in the direction of maximal linear gain due to the feeding strategy and connector attachment. We instead measured in the direction orthogonal to the plane of the antenna and found a gain of $-1.1\pm 0.1$ dBi, compared to a simulated gain of $-1$ dBi in that direction. Type III antennas were simulated with a maximum gain of 1.1 dBi, and we measured a gain of 1.15 dBi for these antennas.

Flexible antennas with compact dimensions and reasonable gain are necessary for UHF-RFID tags, but other components, including an RFIC, matching network, and sensors are needed to create an RFID sensor tag. Conventionally, RFICs and other external components are attached to the antenna’s substrate with solder. However, solder is not compatible with printed flexible antennas because solder reflow temperatures exceed the glass transition temperature of plastic substrates, the resulting joints are very brittle, and many solder compositions erode silver. We developed a mounting procedure for rigid components, illustrated in Fig. 2e, and detailed in the Methods section. Stencil printed silver ink was used for electrical connection; application of the ink through a thin laser-cut stencil as shown in Fig. 2e:i. This provided a small, controlled volume of ink as shown in Fig. 2e:ii, which prevented accidental shorting between neighboring pads when pressure was applied to the chip during bonding, as shown in Fig. 2e:iii. However, silver ink did not provide substantial mechanical stability when the plastic was flexed. Therefore, we used flexible glue for mechanical reinforcement, which also served as encapsulation and protection for the chip.

We measured the impedance of eight RFICs mounted on flexible substrates for input powers from $-20$ to $+ \, 13$ dBm and found impedance that ranged from $1.3-55j\ \Omega$ at low input power to $21-46j\ \Omega$ at high input power. Impedance mismatch between the $50\ \Omega$ antenna and capacitive IC causes power losses which can be characterized by the power transfer coefficient, ($\tau$), defined as:

3 $$\begin{aligned} \tau =\frac{4Re(Z_{ant})Re(Z_{IC})}{|Z_{ant}+Z_{IC}|^{2}} \end{aligned}$$

where $Z_{ant}$ is the antenna’s impedance and $Z_{IC}$ the IC’s impedance. Maximum $\tau$ is achieved when antenna and IC impedance’s are conjugate matched, i.e. $Z_{ant}=Z_{IC}*$. Such matching can be achieved either by tuning the antenna to match the IC^1^ or by using a matching network. We chose to work with an inductor to reduce the imaginary part of the denominator in Eq. (3), which increases $\tau$ and consequently the received power. From measurements of the impedance of both the antennas and the IC, an optimal value of 10 nH was chosen as series inductor. The inductor was attached to the tag using the chip-attachment process described above.

We characterized the performance of the RFID tags by measuring their Return Signal Strength Indicator (RSSI) and read range. Both were measured with an RFID interrogator set up. The interrogator antenna transmited a 30 dBm output signal and received the backscattered signal from the tag. The received power at the reader (which is estimated using the RSSI) is the result of a transmission from the same reader. The received power has experienced two free-space transmissions and one reflection from the tag. Power transmission can be improved by using antennas with higher gain, ensuring they are properly aligned to reduce polarization mismatch, and increasing the power transfer coefficient ($\tau$) between antenna and RFIC, but increasing transmission distance will decrease received power^58^. We measured RSSI for tag-reader distances between 5 cm and 1 m. The reader was unable to make a successful measurement when the RSSI dropped below -40 dBm; the distance where the connection was lost was recorded as the read range.

**Figure 4:**
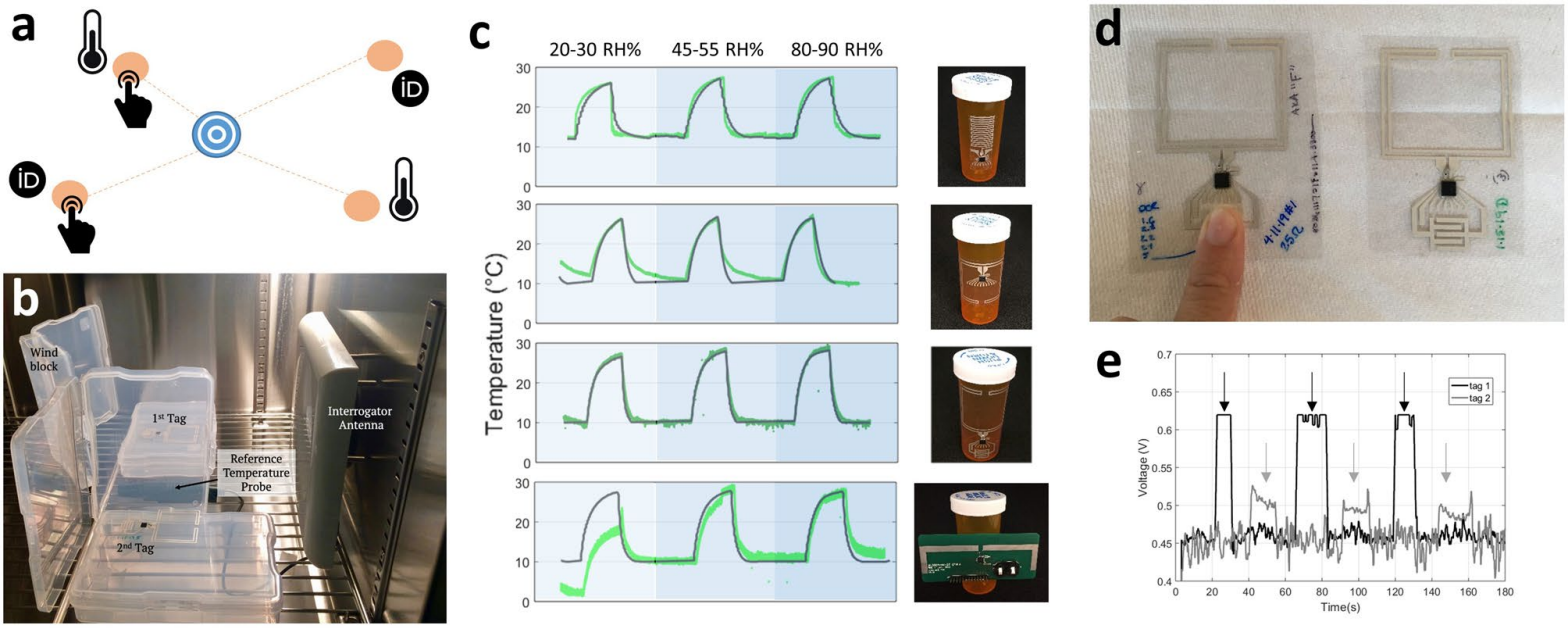
Applications using the printed, flexible, passive tags developed in this study. (**a**) The tags developed in this work, which have a variety of self contained and user-interactive sensors, can form a passive star network with a central active reader. This kind of network only requires the central node to have an energy source, while still allowing for distributed sensing and user-interaction. (**b**) Calibration set-up for measuring temperature in a humidity-controlled environment. Two tags are simultaneously interrogated by the reader antenna. A reference temperature probe is placed between the two tags under test to validate the wireless temperature readouts. (**c**) Results from the temperature measurements show good agreement between wired reference temperature monitor (black) and passive, wireless printed tags (green, top three panels) regardless of humidity. The rigid tag (lower panel) has significant error in low humidity. (**d**) User interacting with a capacitive touch sensor integrated with a type I tag. (**e**) Backscattered sensor data from two type I tags being wirelessly interrogated simultaneously. The user alternated between touching tag 1 and tag 2, and touch events show as elevated voltage readings. This demonstrates that passive networking of user input is feasible using the proposed tags.

Figure 3c shows the RSSI of a screen printed Type I tag and a rigid reference board for transmission distances between 5 and 40 cm. For both tags, RSSI decreased over distance as expected in free-space propagation. The screen-printed Type I tags returned higher RSSI-values than the rigid PCBs closer to the reader (distances < 30 cm) and comparable values at further separation distances (distances 30–40 cm). These measurements were repeated with Type II and III printed tags. For mid-distance transmission (between 15 and 35 cm) the average RSSI of Type I tags was − 30 dBm, for Type II tags it was − 25 dBm, and for type III tags it was − 24 dBm. The rigid reference board had an average RSSI of − 30 dBm over the same range of distances. Type I and II tags’ read range was measured at $40\pm 2.5\hbox {cm}$, while that of Type III tags was much longer, $90\pm 2.5\hbox {cm}$, and the read range of the reference board was 60 cm. This data is also summarized in Supplementary Table 2.

Type III antennas were designed to be customized after chip attachment. As-printed antennas include 18 two-centimeter segments and resonate below 915 MHz. Meanders can be trimmed to shift the resonance to the desired frequency. This option makes Type III antennas compatible with RFID standards in different global regions and allows each antenna to be optimized for the impedance of the RFIC. We found that trimming three segments from the meandered antenna led to the highest RSSI at 915 MHz.

We used tags with all three antenna designs to wirelessly transmit sensor data. We first demonstrated transmission of sensor data using the RFIC’s on-board temperature sensor. Figure 4b shows the setup in an environmental chamber used to calibrate the temperature sensor of the screen-printed RFID tags. The temperature and humidity inside the chamber were controlled, and a commercial wired sensor was used for reference. The interrogator antenna and two tags were placed inside the chamber, separated by about 20 cm. The temperature was modulated from cool (10 °C) to warm (25 °C) at three different humidity levels. In humid environments, many polymers absorb water, which changes their material properties and can impact the performance of devices fabricated on these substrates. The sensitivity to humidity depends on the specific material. Figure 4c shows the temperature measured by the RFID tags in green compared to the wired reference temperature measurements in black. Each of the three antenna types, as well as a rigid reference board, were tested. The printed tags showed increases and decreases in temperature that aligned very well with the measurements of the references probe, once the measure data were filtered for outliers. There was an average relative error of 0.15% on the temperature measurements. We found that the tags were able to accurately measure temperature regardless of the humidity. At high humidity, the data from printed tags did have spikes due to irregularities in the wireless communication. In general, these errors were fairly uncommon: 6% of the measurements for tag Type I and lower percentages for the other tags. Our calibration measurements showed that our strategy to use the RFIC’s temperature sensor with our fabrication technique led to reliable temperature measurements in different humidities.

Sensor tags can be made more versatile by integrating different types of external sensors. We chose to integrate a printed capacitive touch sensor to illustrate this possibility. Capacitive touch senors are composed of interdigidated electrodes which have some capacitance between them. The fringing fields extend out of the plane of the electrode, making them sensitive to the environment nearby^59–61^. When a finger, whose dielectric constant is much greater than that of air, is placed on the sensor, the capacitance of the sensor increases. The sensing capacitor was configured as a voltage divider with a reference capacitor whose capacitance did not change when the sensing capacitor was pressed. The RFIC measured the voltage on its analog input ports. Figure 4d shows two of the fully printed sensor tags with capacitive touch sensors at the bottom. The sensing capacitor and reference capacitor were made of printed silver traces. These patterns were incorporated into the antenna screen layout, resulting in a complete sensor tag that was printed in a single pass.

Figure 4e shows the transmitted voltage detected by the external voltage sensor of two different screen-printed RFID tags that simultaneously transmited that voltage using UHF-RFID backscattering. The touch events were clearly visible in the transmitted data. When the tags were not touched, the voltages were $0.46\pm 0.004~V$ and $0.45\pm 0.02~V$ for tags 1 and 2, respectively. During touch events those voltages increased to $0.62\pm 0.006~V$ and $0.50\pm 0.01~V$ for tags 1 and 2, respectively. These increases and changes of standard deviation were significantly larger than the RFIC’s sensitivity of 0.01 V. However, we did observe tag-to-tag variation in the voltage divider. A tag-by-tag calibration of the cap-touch sensor would be required if the read-out system had no learning abilities in order to distinguish between touch and non-touch events.

## Discussion

Our work builds on and brings together advancements in the fields of antenna miniaturization, printed and flexible UHF-RFID tags, and printed capacitive touch sensors. Antenna miniaturization enables compact sensor tags^1,49^. Printed UHF-RFID enables integration with everyday objects or packaging in supply chain management, either by printing the antenna on a flexible substrate which is attached to the object^15,16^, or by printing directly on the packaging^14^. Printing miniaturized antennas, demonstrated in this work, enables their integration with a greater variety of objects. Earlier demonstrations of UHF-RFID sensor tags use commercial sensors and circuit elements^15^. Each component is rigid, protrudes from the plane of the print, and must be attached to the flexible substrate. Printed sensors, including printed capacitive touch sensors^59–61^ give the sensor the same properties as the antenna: physical flexibility, ease of manufacture and integration. However, these printed sensors are only useful when integrated with reading electronics and a communication link. Here we have shown that combining a compact antenna design with printing of the antenna and external sensor can produce a complete sensor tag that can be more easily integrated with objects.

Screen printing offers many benefits for the production of RFID sensor tags. Screen printed antennas’ thickness and conductivity minimize losses, their gain and bandwidth match simulation, and they are functional while flexed. Screen printing also provides sub-100 μm resolution which is needed to create landing pads for rigid IC’s and surface mount components. Some external sensors can be seamlessly integrated into the RFID tag using screen printing. It is also a scalable, additive manufacturing technique.

However, screen printing is not well suited for prototyping antenna designs because each design requires a unique screen. Screens are typically ordered from a commercial manufacturer. A fabrication technique that allows researchers to modify the designs in the research lab is advantageous to the design process. From our characterization of different printing techniques, stencil printing emerged as a promising model for screen printing. Stencil printed samples’ thickness, impedance, and S-parameters are comparable to those of screen printed antennas. Inkjet printing produces antennas whose properties are markedly different from screen printed antennas.

There are some printed, flexible UHF-RFID tags demonstrated in literature^14–16^. However, these tags use an antenna footprint that elongates the tag and not much information on the used printing technique, nor the coupling between the RFIC and the antennas is provided. In comparison to other printed UHF-RFID systems that use the same IC^14,15^, we use antennas that have a smaller footprint, see Fig. 1b, test different antenna printing methods, see Fig. 2a,b, and d, outline a methodology for fabrication, see Fig. 2c, and demonstrate performance of the antenna under bending conditions, see Fig. 2e. Screen-printed UHF-RFID tags using the same RFID IC were previously demonstrated on polyimide Kapton^15^ and on cardboard^14^ with the purpose of chemical sensing and package tracking, respectively. The antennas used in these references have larger dimensions and a lower a gain of 0.66 dBi than measured in this work.

Since^14–16^ use the same IC, they should also be able to measure temperature. However, a calibration of the temperature sensor is not provided in those references. A High-Frequency (HF) RFID tag with a printed thermistor is presented in^22^. Those tags show a similar sensitivity and accuracy to the tags presented in this paper and use a similar footprint (90 x 70 mm^2^). The main alternative to using an temperature sensor integrated in the IC would be to use a printed temperature sensor on the flexible substrate^62–64^. Such a printed sensor could be compact and integrated into the antenna^64^, but it will inevitably use more space on the tag than the chip’s on-board temperature sensor.

Printed, interdigitated capacitive touch sensors have been demonstrated in literature^59–61^ and alternative printed, stacked architectures exist^65^. However, these capacitive touch sensors are only demonstrated using rigid (printed) boards for readout and no networking using multiple tags has been demonstrated.Wireless systems with user-interaction through capacitive touch sensors have been proposed in literature^66–69^, but such systems are either not passive^66,69^, do not transmit the sensor data directly^68^, use a rigid external board for processing^69^, or are not printed on a flexible substrate^66,67^.

The passive RFID sensor tags can be connected in a star network in which only the central reader is connected to a power source. The passive nodes enable distributed sensing with a variety of sensor types, including touch sensors which allow for user-interaction.

## Conclusions

We developed a screen-printed, flexible, wireless temperature sensor tag using passive UHF RFID using printed, flexible dipole antennas. These miniaturized antennas featured moderate gain and small linear dimensions. Different printing techniques were investigated to fabricate this antenna. Screen-printing the antenna was found to lead to the most efficient antenna, with stencil printing using a disposable stencil as a good precursor for screen-printing. Inkjet printing did not lead to antennas with sufficient efficiency. The antenna was coupled with a commercially available UHF-RFID generation 2 RFIC, which was mounted on a flexible substrate using a mounting strategy using blade-coated silver ink. The RFIC’s internal temperature sensor was calibrated in an environmental chamber, demonstrating the tag’s passive, wireless functionality. Additionally, a printed interdigitated capacitive-touch sensor was added to the tag. We demonstrated that this passive sensor can be read out for multiple tags simultaneously over the network.

## Methods

### Antenna printing

*Printing materials* Tags and antennas were fabricated using different printing techniques on plastic substrates. All samples were printed on a 125 μm-thick polyethylene naphtalate (PEN) substrate (Q65HA from Teijin Dupont Films, Wilmington, DE, USA).

*Inkjet printing* Antennas were printed with DGP 40LT-15C silver ink (Advanced Nano Products, Sejong, Korea) using a Dimatix DMP-2850 inkjet printer (Fujifilm Dimatix, Santa Clara, CA, USA) with a 10-pL cartridge. Samples were printed with a drop spacing of 25 μm and a platen temperature of 52 °C during printing, and annealed in a vacuum oven for 1 h at 140 °C.

*Spray coating* Patterns were defined using laser cut stencils. The stencils were cut from adhesive Kapton film (Dupont) using a laser cutter (Universal Laser Systems Inc, Scottsdale, AZ, USA). Silver ink designed for spray coating, PSPI-1000 ink (NovaCentrix, Austin, TX, USA), was applied by hand with an aerosol brush. After printing, the samples were annealed at 130 °C for 15 mins.

*Stencil printing* The stencils for stencil printing were prepared in the same way as the stencils for spray coating. Silver ink, 126-33 extremely conductive silver ink (Creative Materials, Ayer, MA, USA), was applied using a razor blade held in contact with the stencil. Samples were annealed on a hot plate at 130 °C for 10 min, then the adhesive stencil was removed.

*Screen printing* The screens were ordered from NBC Meshtech (Bavaria, IL, USA). Screen printed samples used the same ink and annealing conditions as stencil printed samples.

*Antenna profiles* Thickness and roughness of the traces produced by each technique were measured with a Veeco Dektak MG stylus profilometer.

### Chip attachment, impedance matching

The rigid components, which were the RFIC, an SL900A by AMS (ams AG, Premstaetten, Austria), 0603-form inductors, and 0603-form capacitors were attached to printed traces on flexible substrates in a two step process. First, Creative Materials 125-13H silver ink was applied with a razor blade through a kapton stencil, as shown in Fig. 2e. The stencil is 50 μm thick non-adhesive kapton film (DuPont), and the features were cut with a laser engraver. Stencil and substrate were held in place with a gel-pack backing layer. A pick-and-place die bonder was used to align and mount all rigid components on the wet ink. The ink was cured at 180 °C for three minutes. Finally, both the RFIC and the passives were coated with a few drops of Loctite 4902 flexible super glue, which dried at room temperature for several hours.The attachment process was optimized for stability and conductivity. We tested six types of conductive adhesive materials by using them to attach zero-ohm resistors to screen printed traces. A screen printable silver ink was selected for its high conductivity, compatibility with printed silver traces, and stability when the substrate was slightly flexed. The materials tested and their limitations are summarized in Supplementary Table 2.

### Antenna design and simulation

The antennas were designed using numerical simulations in the finite-difference time-domain (FDTD) simulation software Sim4Life (ZMT, Zürich, Switzerland). The dipoles were developed based on the designs presented in^29^. The dimensions of the dipole traces were adapted to achieve a target power reflection coefficient < − 10 dB in the UHF RFID frequency band (902–928 MHz in the USA) in reference to 50 Ohms. The meandered monopole was designed to be compatible with the stencil printing methodology. Therefore, minimum trace thickness and inter-trace spacing were kept at 1 mm. The meander width and number of meanders were varied in order to achieve a power reflection coefficient < − 10 dB in the UHF RFID frequency band. The conductive traces were modeled as conductive sheet with $\sigma$ equal to the conductivity of the conductive ink used for screen printing ($\sigma = 5\times 10^6 S/m$) and the PEN substrate as a brick with $\epsilon _r$ = 3.5, $\sigma$ = 0 S/m, and thickness = 125 micrometer. Conductive traces were modeled with a maximal grid step of 1 mm in the FDTD algorithm. The substrate was modeled with a single step of 125 micrometer in the transverse dimension and maximal steps of 1 mm in the other two dimensions. The antennas were fed a Gaussian pulse with center frequency 1 GHz and bandwidth 1 GHz using a voltage source. The voltage and current on the antennas are monitored using the simulation software and the simulation is terminated once those quantities reach a steady-state solution.

### Antenna characterization

During all measurements the antennas were directly connected to an SMA cable using the 292-64A-06 connector. Voltage reflection coefficient ($S_{11}$) measurements of antennas printed using different techniques were executed using a VNA (Agilent N5242A, PNA-X, Santa Clara, CA, USA). All $S_{11}$ measurements were executed in reference to 50 $\Omega$. Antenna gain measurements of antennas printed using different techniques were executed using two-port measurements with the same VNA. The VNA registered two-port S-parameters of two antennas mounted at a fixed distance of 61 cm. S-parameter were averaged over 50 sweeps from 500 to 1500 MHz with 1001 frequency steps of 1 MHz and were then used in the three-antenna method^70^ to determine antenna gain. During these measurements, all antennas were placed parallel with their printed sides facing each other in a mirrored configuration. Antenna gain as function of $(\theta ,\phi )$ and polarization were measured with the same VNA in the same setup as the antenna gain measurements. During these measurements, one antenna was kept static, while the second antenna in the transmission measurement setup was rotated in either the azimuth angle ($\phi$) or the elevation angle ($\theta$) in order to obtain the dependency of $G_{\phi }$ and $G_{\theta }$ on both components, resulting in a radiation pattern. Antenna bending measurements were executed using the screen-printed antennas and the same VNA with the same configuration as the measurements described above. During these measurements, one antenna was kept static, while the second was bent around it’s middle horizontal and vertical axes of the antenna as shown in Fig. 2d.

### Over-the-air RF tag performance testing

The RFID tags were placed in front of a commercially available RFID reader system (Thingmagic Sargas, Trimble, Sunnyvale, CA, USA) connected to a circularly polarized RFID reader antenna (MT- 262024/TRH/A/K from MTI Wireless Edge) with 30 dBm output power. The tags were placed with their printed surface parallel to the surface of the RFID-reader antenna at a distance of 5 cm and were moved away from the reader antenna in the direction orthogonal to the reader antenna in steps of 5 cm. At every step in separation distance, the reader registered the received signal strength indicator (RSSI) value that was returned by the tag. RSSI measurements were collected using Sargas’s web GUI. Twenty RSSI values were registered at every location and determined the average (in decibels) RSSI over distance. The same measurements were executed using two reference PCBs, produced by the manufacturer of the SL900A (Fig 1c). For the tags of Type III, one length of the meander (2 cm) was cut off with scissors, then the the RSSI-vs-distance measurements were repeated. This process was repeated 6 times. From this information, an optimal antenna length was found.

### Over-the-air calibration of tag temperature sensor

The SL900A contains an internal temperature sensor that performs relative temperature measurements. The same interrogator antenna described above and two screen-printed tags were placed in an environmental chamber (Associated Environmental Systems, Ayer, MA, USA). The chamber went through three temperature cycles (10 °C to 30 °C and lowered again to 10 °C) at different relative humidities (RH): low humidity (10 to 30% RH), medium humidity (45 to 50% RH), and high humidity (80 to 90% RH). A reference measurement inside the chamber was executed using a wired probe (RH520A, Extech Instruments, Boston, MA, USA). The same measurements were executed for each type of tag and the rigid reference boards, see Fig. 4c.

### Capacitive touch sensor design

The capacitive touch sensor was made with five fingers, with overlap length of 9 mm, width of 1.5 mm, and gap spacing of 1 mm. The layout is included in Supplementary Figure A.1a. These capacitors had an average capacitance of $1.33\pm .04$ pF when not touched. Capacitance increased up to 24 pF when touched, depending on the force of the touch. The capacitor is measured by the RFIC in a voltage-divider set up. We used the small stray capacitance between two printed traces as the reference capacitor in the voltage divider.

### Over-the-air tag capacitive-touch read-out

During these measurements, the tags were laid on a brick of styrofoam at 20 cm from the same UHF-RFID reader used in the previous section. The tag’s voltage sensor 1 was interrogated continuously using the wireless channel and a time series of the measured voltage was stored on the reader’s side. Sensor measurements were recorded using Mercury API java scripts from ThingMagic. The tags were then touched periodically on the capacitive touch sensor. These measurements were executed for two tags simultaneously, in order to show the networking abilities of the UHF-RFID setup using our screen-printed tags.

## Supplementary information

Supplementary Information.
