# Supplementary material for: Printed, flexible, compact UHF-RFID sensor tags enabled by hybrid electronics
Source: Sci Rep. 2020 Oct 6;10:16543. doi: 10.1038/s41598-020-73471-9 (PMC7538943; doi:10.1038/s41598-020-73471-9)
Supplement: Supplementary file 1 — Supplementary Information. [file 41598_2020_73471_MOESM1_ESM.pdf]

Supplementary
Compact, Passive UHF-RFID Sensor Tags Enabled by Flexible Hybrid Electronics

Carol Baumbauer, Matthew G. Anderson, Jonathan Ting, Akshay Sreekumar, Jan M. Rabaey, Ana C. Arias, and Arno Thielens

A Antenna and Tag Dimensions

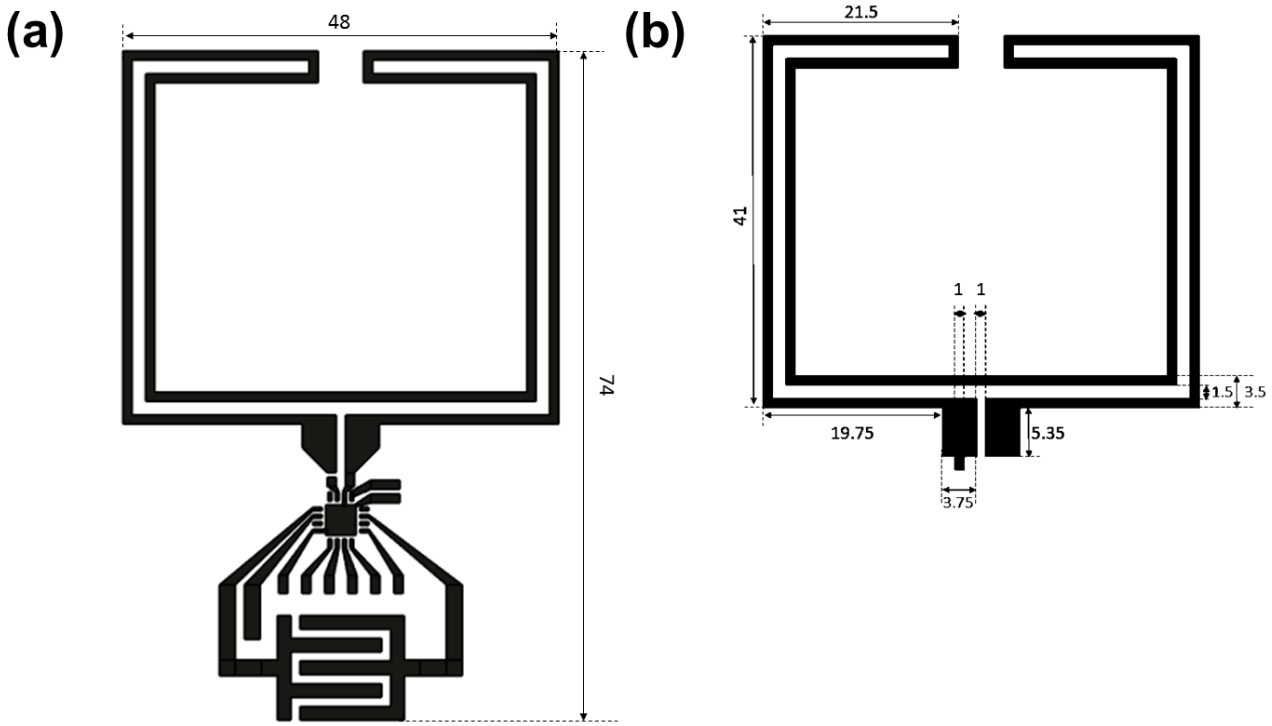

Figure A.1. Dimensions of tag and antenna Type I in mm (a) Tag type I and (b) Antenna Type I.

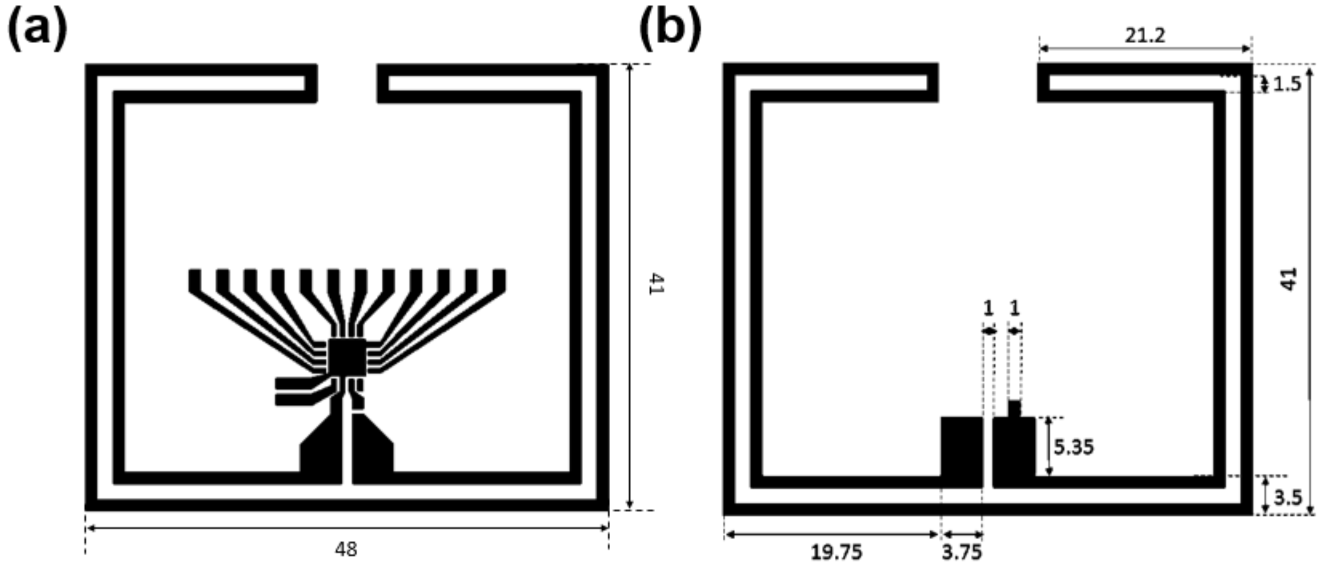

Figure A.2. Dimensions of tag and antenna Type II in mm: (a) Tag type II and (b) Antenna Type II.

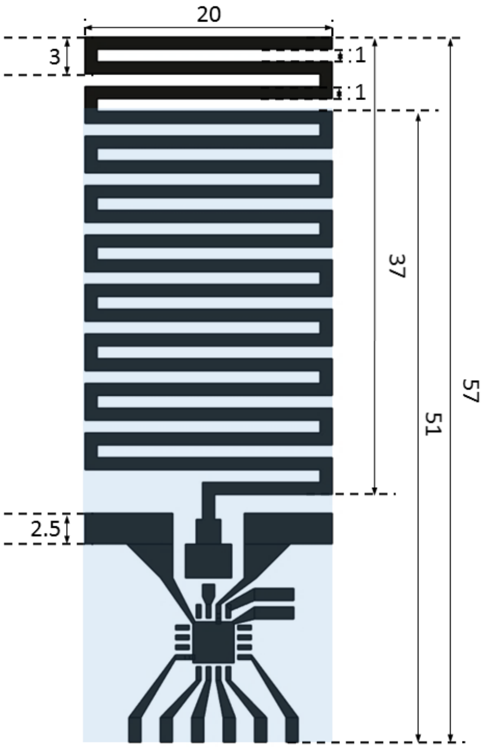

Figure A.3. Dimensions of print and tag Type III in mm. The area shaded in blue shows the UHF-RFID tag. The three upper meanders are included in the screen-print, but are not included in the final tag.

B Component Attachment Materials

Material	Limitations
Ted Pella Conductive Silver paint (PELCO # 16062)	High Resistive losses Not printable, brittle
MG Chemicals Silver Conductive Epoxy Adhesive	High resistve losses, Not printable, brittle
Creative Materials 125-13H Silver Screen Printable Ink	Good conductivity, Moderately flexible
Creative Materials 120-07: Fine Features Screen Printable Ink	Good conductivity, Moderately flexible
Creative Materials 126-33: Extremely Conductive Screen Printable Ink	Good conductivity, Moderately flexible
Koki M742 Low Melting Point Sn-Ag-Bi Solder Paste	Leaches silver ink, High conductivity Very brittle

Table 1. Properties of materials tested for component attachment

C Over-the-air RF Tag Performance Testing

	Antenna Type	Feed Type	Flexible [y/n]	Size [cm x cm]	Read Range [cm]	(P_r)† [dBm]
Type I	Folded Dipole	external	y	48 x 46	40	-30 ± 5
Type II	Folded Dipole	internal	y	41 x 46	40	-25 ± 2
Type III	Monopole	external	y	51 x 20	90	-24 ± 9
Reference	Dipole	external	n	117 x 23	60	-30 ± 3

†Average RSSI between 15 and 35 cm ± standard deviation

Table 2. Comparison of rigid and printed tag performance

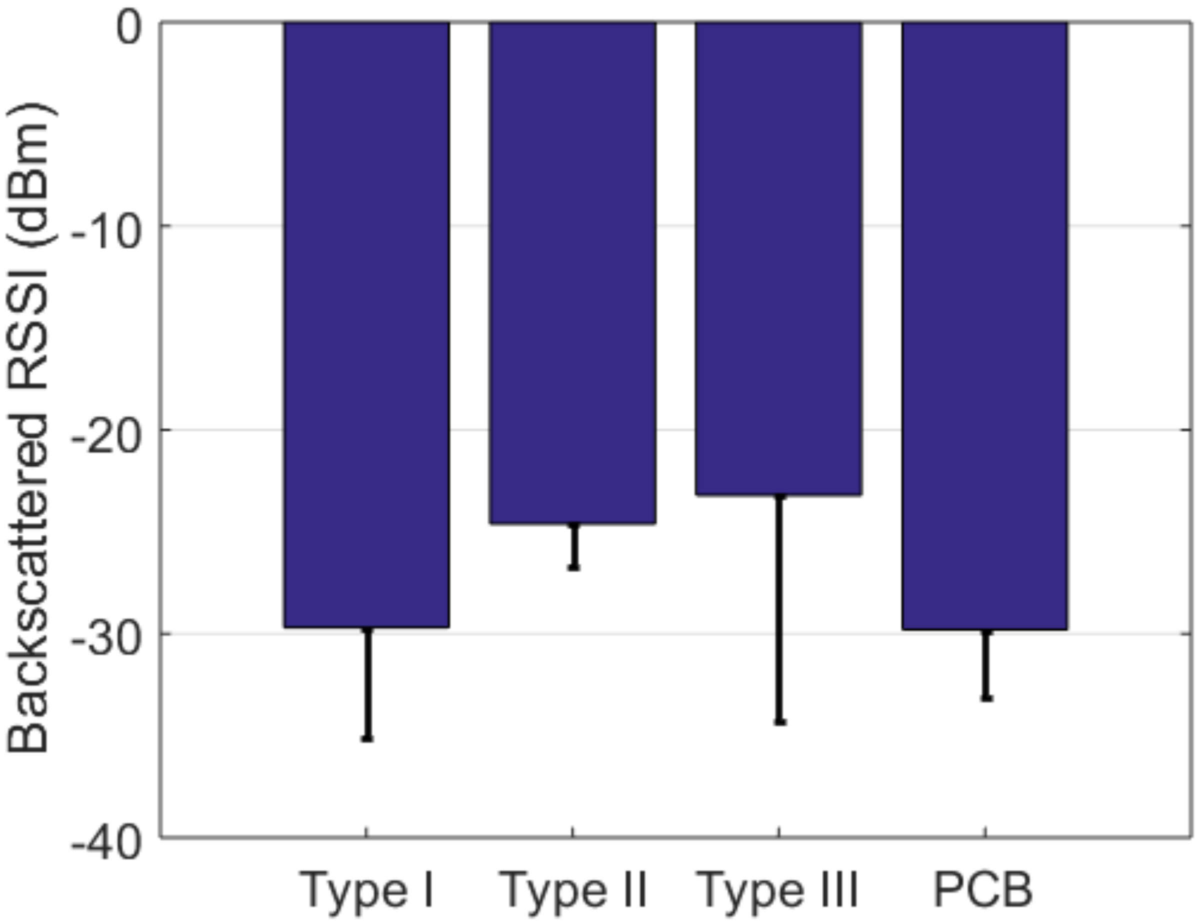

Figure C.1. Received Signal Strength indicator (RSSI) averaged over separation distances from 15 cm to 35 cm for the three studied screen-printed tag types and the rigid PCB shown in Fig 1 (c). The bars indicate the mean back scattered signal strength over those distances, while the whiskers indicate the expanded uncertainty on the measurements consisting out of the variation over distance and the variation at fixed distance in RSSI.
